# Active structural acoustic illusions

**DOI:** 10.1038/s41598-020-66398-8

**Published:** 2020-06-17

**Authors:** Daniel Eggler, Nicole Kessissoglou

**Affiliations:** School of Mechanical and Manufacturing Engineering, The University of New South Wales, Sydney, 2052 Australia

**Keywords:** Engineering, Mechanical engineering, Acoustics

## Abstract

We present active manipulation of the structural vibrations of an elastic body to generate an acoustic illusion. The resultant illusion misrepresents the nature, size and number of objects in the exterior acoustic domain. We demonstrate our technique, herein termed active structural acoustic illusion, using an elastic cylindrical shell. The radial motion of the shell at its cavity resonance frequencies is actively modified using localised mechanical forces. Acoustic illusions are generated to resemble the scattered acoustic field by one or more rigid cylinders of different size and location.

## Introduction

Illusions are an alternative to cloaking targets from detection, by generating misrepresenting information about a target. The first optical illusions were passively achieved utilising metamaterials that altered the wavefield propagation pathways. Metamaterial shells comprising complementary media layers incorporating double effective negative parameters have been able to modify information about a given target such as size^1,2^, shape^3–5^, location^6^, and material properties^7,8^. Electromagnetic illusions have also been implemented using active approaches, whereby control sources were employed to actively modify the electromagnetic field to the desired optical illusion^9–12^.

The first acoustic illusion was presented by Kan *et al*.^13,14^ using a metamaterial shell comprising positively indexed anisotropic material. They numerically and experimentally showed that scattered waves from a cylinder appeared as scattering from a rectangular prism. Similarly, Liu and He^15^ exchanged the scattering potential of a given object with that of a desired illusion by utilising positively indexed isotropic materials. More recently, passive acoustic illusions have been achieved using a tunable metasurface^16^, an enclosed device with subwavelength thickness^17^, and a multi-folded transformation method^18^.

In contrast to passive approaches for illusions that are restricted to predetermined scenarios, active techniques allow for parameter adjustments to be made in real-time. Börsing *et al*.^19^ employed a distribution of monopole sources on a rigid surface and mapped the physical boundary conditions of the rigid surface to a virtual environment. The incident field was suppressed before interacting with the rigid surface whilst the desired illusion field from the virtual environment was simultaneously generated. Rajabi and Mojahed^20^ applied piezoelectric patches to an elastic spherical body to actively modify the surface shell velocities such that the resultant acoustic field resembled that of a larger sphere or a sphere of different material composition. Whilst the aforementioned acoustic illusions employed active methods, the proposed methodologies do not lend themselves to practical realisation due to the exclusion of an active controller. The current authors recently employed conventional active noise control techniques to actively modify the acoustic field of a rigid cylinder arising from scattering by an incident field^21^. Acoustic illusions were generated to misrepresent the size and location of the rigid cylinder. The ability to replace the acoustic field arising from scattering by a rigid body with a different incident field was also demonstrated.

The field of active noise and vibration control is well established (for example, see^22,23^) and presents a feasible approach for practical implementation of acoustic illusions. Active structural acoustic control (ASAC) that combines active noise control (ANC) and active vibration control (AVC) has been demonstrated to effectively reduce sound radiation from vibrating structures. Structural actuators are employed to actively manipulate a vibrating structure, where the optimal control forces are obtained from minimisation of a cost function based on the sound radiation. Early work using ASAC investigated the use of control forces to attenuate structure-borne sound from uniform rectangular and circular plates^24–26^, and plates with discontinuities such as a stiffening rib^27^. ASAC has also been successfully implemented to minimise the radiating sound from elastic cylindrical shells^28–30^. Advantages of ASAC compared to ANC include robust performance and reduced control effort^24,28,30,31^.

We herein present acoustic illusions of an elastic structure based on an ASAC approach. To this end, control inputs are directly applied to actively manipulate the structural vibrations of a cylindrical shell that efficiently radiated sound. Our technique, herein termed active structural acoustic illusion, minimises a cost function based on a two-stage process that replaces the sound field associated with the original object (a cylindrical shell) with an acoustic illusion corresponding to the sound field associated with one or more rigid objects of different size and location. Our control method forms a platform for practical realisation of acoustic illusions using well-established active control techniques. Employing an adaptive controller allows for variation in frequency and physical system behaviour to be accommodated, thus yielding the ability to misrepresent the nature of an object being perceived in real time.

## Methods

### Active control configuration and theory

The active control system comprises a multichannel control configuration with *W* control forces and *L* error sensors. We minimise a quadratic cost function based on a feedforward adaptive least-mean-square algorithm^32^. The first stage is the removal of the acoustic field at the error sensor locations (sound cancellation). The second stage generates the desired acoustic illusional field (sound reproduction). Our control approach is applied to a two-dimensional elastic cylindrical shell. The physical system responses associated with the original and illusional objects must be established prior to implementing the control process. The mathematical formulation to describe the acoustic fields of an elastic cylindrical shell (original object) and illusion object (one or two rigid cylinders) is provided in the Supplementary Material^33,34^. The quadratic cost function corresponding to the sum of the squared acoustic pressures at each error sensor location is given by

1 $$J={{\bf{e}}}^{{\rm{H}}}{\bf{e}},$$

where **e** is a vector denoting the acoustic field at the error sensors and H denotes the Hermitian transpose operator. The error vector can be expressed as

2 $${\bf{e}}={{\bf{p}}}_{{\rm{structural}}}+{\bf{Zq}}-{{\bf{p}}}_{{\rm{illusion}}},$$

where ${{\bf{p}}}_{{\rm{structural}}}$ denotes the shell’s structure-borne sound pressure arising from excitation due to a plane wave (Eq. (3) in the Supplementary Material) or a monopole source (Eq. (4) in the Supplementary Material). **Z** is an $L\times W$ matrix of complex transfer functions representing the radiated field due to *W* control forces (Eq. (7) in the Supplementary Material is the sound field for single force excitation of the shell). ${{\bf{p}}}_{{\rm{illusion}}}$ is a vector representing the acoustic pressure of the desired illusion at the *L* error sensor locations (Eq. (13) in the Supplementary Material represents the acoustic pressure at a single error sensor). **q** is the vector of *W* optimised complex control force amplitudes. Substituting Eq. (2) into Eq. (1), differentiating the resulting expression with respect to the real and imaginary components of **q** and equating to zero, that is, $\partial J/\partial {\bf{q}}=0$, the optimised control force amplitudes can be obtained as

3 $${\bf{q}}=-{[{{\bf{Z}}}^{{\rm{H}}}{\bf{Z}}]}^{-1}{{\bf{Z}}}^{{\rm{H}}}({{\bf{p}}}_{{\rm{structural}}}-{{\bf{p}}}_{{\rm{illusion}}}).$$

The inversion of the matrix ${{\bf{Z}}}^{{\rm{H}}}{\bf{Z}}$ is carried out using a Moore-Penrose pseudoinverse function. Since ${{\bf{Z}}}^{{\rm{H}}}{\bf{Z}}$ is a Hermitian, positive-definite matrix, the inverse always exists and is unique^22^. The inversion is well-behaved provided the number of error sensors is greater than the number of control sources as this ensures the least-mean-squares problem is overdetermined. The aforementioned active control method utilises *a priori* knowledge of the incident field. There are limited strategies to overcome this issue, however the ability to measure and deduce key information about the incident field can be found in^35–37^.

**Figure 1:**
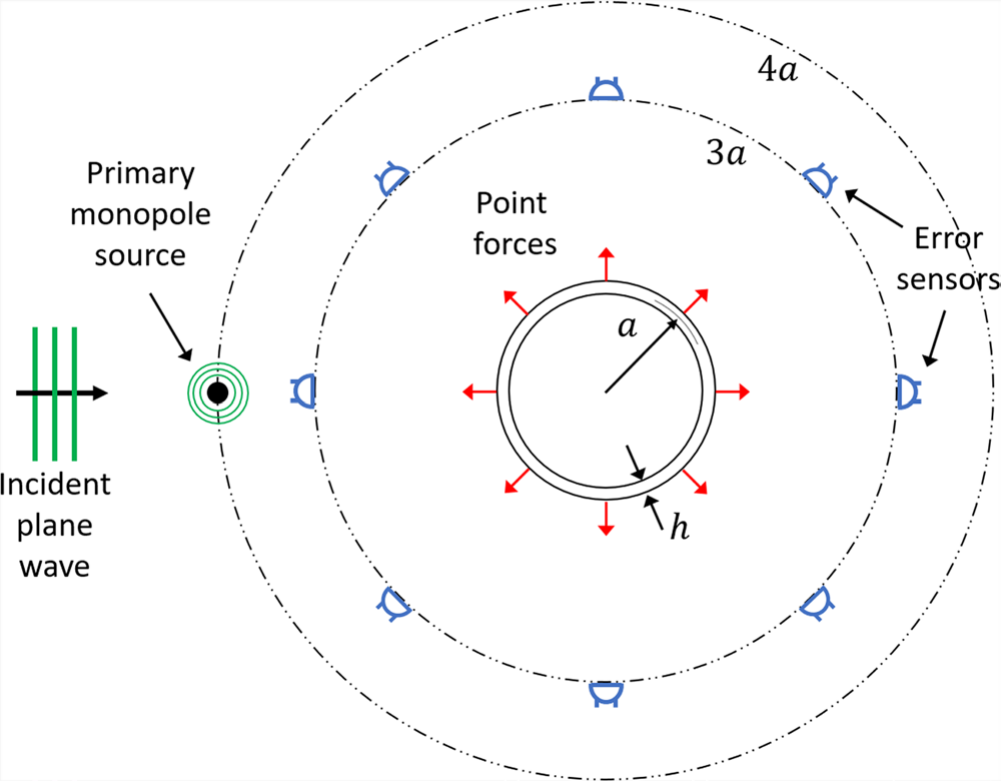
Control arrangement for an elastic cylindrical shell of mean radius *a* and thickness *h*. Point forces are located on the surface of the shell with equispaced error sensors circumferentially distributed around the shell at 3*a*. The primary acoustic field is due to an incident plane wave or monopole source located 4*a* upstream of the shell.

We demonstrate our active structural acoustic illusion technique on a 2D elastic cylindrical shell of thickness *h* = 4 mm and radius *a* = 0.6 m measured to mid-plane thickness, as shown in Fig. 1. The shell material is polyvinyl chloride with properties corresponding to density 1300 kg/m^3^, Young’s modulus 2.9 GPa and Poisson’s ratio 0.3. The interior and exterior fluid media correspond to air with density and sound speed of $1.225\,{{\rm{kg}}/{\rm{m}}}^{3}$ and 343 m/s, respectively. Localised control forces were distributed equally around the shell. Similarly, error sensors were distributed circumferentially around the shell at a radial distance of 3*a* from the shell centre.

## Results

All results were generated using MATLAB. The number of control forces and error sensors implemented in the control configuration for each illusion case study were determined using a similar procedure outlined in ref. ^38^ based on a percentage error function given by

4 $$\Delta =\frac{||{{\bf{p}}}_{{\rm{illusion}}}|-|{{\bf{p}}}_{{\rm{controlled}}}||}{|{{\bf{p}}}_{{\rm{illusion}}}|}\times 100 \% ,$$

where ${{\bf{p}}}_{{\rm{controlled}}}$ is a vector of the actively modified acoustic pressure at the error sensors. The number of control forces and error sensors for each illusion were obtained for the minimum value of $\Delta$.

### Single body illusions

**Figure 2:**
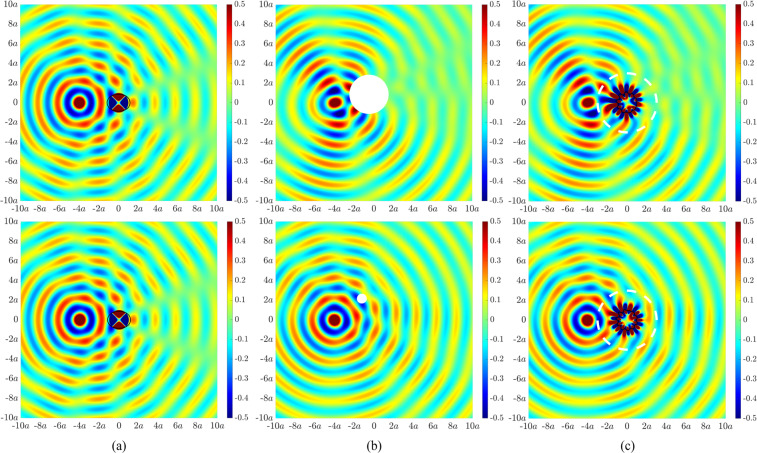
Acoustic pressure fields (in Pascal) under monopole source excitation at 283.4 Hz for (**a**) an elastic cylindrical shell of radius *a*, (**b**) (top) a rigid cylinder of radius $2a$ centred at a radial distance of *a* and an angle of $120^\circ$ to the horizontal axis from the centre of the elastic shell, (**b**) (bottom) a rigid cylinder of radius $0.5a$ centred at a radial distance of $2.5a$ and an angle of $120^\circ$ to the horizontal axis from the centre of the elastic shell, (**c**) an elastic cylindrical shell of radius *a* with an actively generated acoustic field to resemble the scattered acoustic field in (**b**). The dashed white line represents the error sensor perimeter at $3a$.

We first demonstrate results for single body illusions misrepresenting the nature, size and location of the cylindrical shell. Figure 2(a) presents the acoustic field arising from a monopole source located at $4a$ upstream of the cylindrical shell at an excitation frequency of 283.4 Hz. This frequency corresponds to an internal (0,2)-resonance within the shell cavity as indicated by the two radial nodal lines^39^. Significant scattering can be observed downstream of the shell. Figure 2(b) shows the desired acoustic illusional fields associated with larger and smaller rigid cylinders that are shifted from the centre of the original cylindrical shell. The top panel corresponds to the scattered acoustic field for a rigid cylinder of radius 2*a* centred at a radial distance of *a* and an angle of 120° to the horizontal axis from the centre of the shell. The bottom panel corresponds to the scattered acoustic field for a rigid cylinder of radius $0.5a$ centred at a radial distance of $2.5a$ and an angle of 120° to the horizontal axis from the centre of the shell. Greater asymmetric acoustic scattering is observed for the larger cylinder. The active structural acoustic illusions are presented in Fig. 2(c), in which the radial motion of the shell in Fig. 2(a) is actively modified such that the structure-borne pressure yields the acoustic field in Fig. 2(b). Beyond the radial location of the error sensors (indicated by a white dashed line), the actively generated acoustic field matches the desired illusional field. The control configuration utilised 19 forces and 35 error sensors for Fig. 2(c) (top), and 21 forces and 87 error sensors for Fig. 2(c) (bottom).

### Multiple body illusions

**Figure 3:**
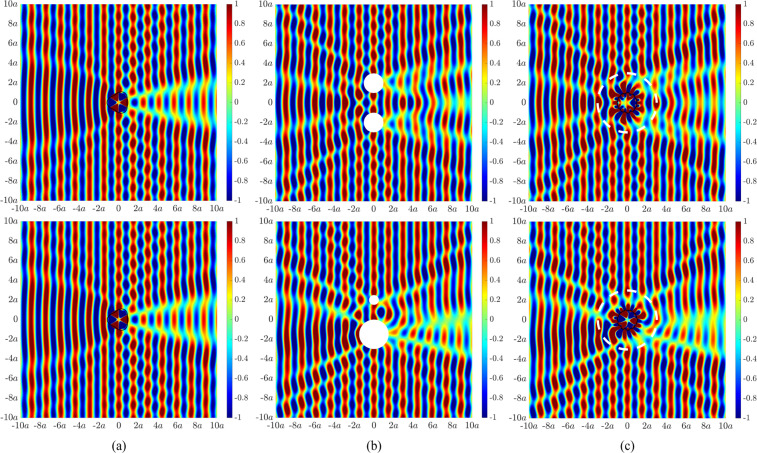
Acoustic pressure fields (in Pascal) under plane wave excitation at 387.6 Hz for (**a**) an elastic cylindrical shell of radius *a*, (**b**) (top) two rigid cylinders of radius *a* located directly above and below the centre of the shell at a radial distance of 2*a*, (**b**) (bottom) two rigid cylinders of radius $0.5a$ and $1.5a$ located directly above and below the centre of the shell at a radial distance of 2*a* and $1.5a$ respectively, (**c**) an elastic cylindrical shell of radius *a* with an actively generated acoustic field to resemble the scattered acoustic field in (**b**). The dashed white line represents the error sensor perimeter at 3*a*.

We now generate active illusions which are capable of misrepresenting the size, location and number of objects. Figure 3(a) shows the acoustic field due to an incident plane wave impinging on the elastic cylindrical shell at an excitation frequency of 387.6 Hz. This frequency corresponds to an internal (0,3)-resonance within the shell cavity as indicated by the three radial nodal lines^39^. The desired acoustic field corresponds to acoustic scattering by two rigid cylinders. The first case comprises two cylinders each of radius *a* and located 2*a* directly above and below the centre of the shell, resulting in symmetric scattering (Fig. 3(b), top). The second case comprises a rigid cylinder of radius $0.5a$ located 2*a* directly above the centre of the shell in conjunction with a rigid cylinder of radius $1.5a$ located $1.5a$ directly below the centre of the shell, resulting in asymmetric scattering (Fig. 3(b), bottom). Figure 3(c) presents the acoustic illusions whereby the actively modified shell displacement has effectively reproduced the scattered acoustic field arising from multiple objects. The control configuration utilised 21 forces and 61 error sensors for Fig. 3(c) (top), and 21 forces and 50 error sensors for Fig. 3(c) (bottom).

### Limitation of active structural acoustic illusions

**Figure 4:**
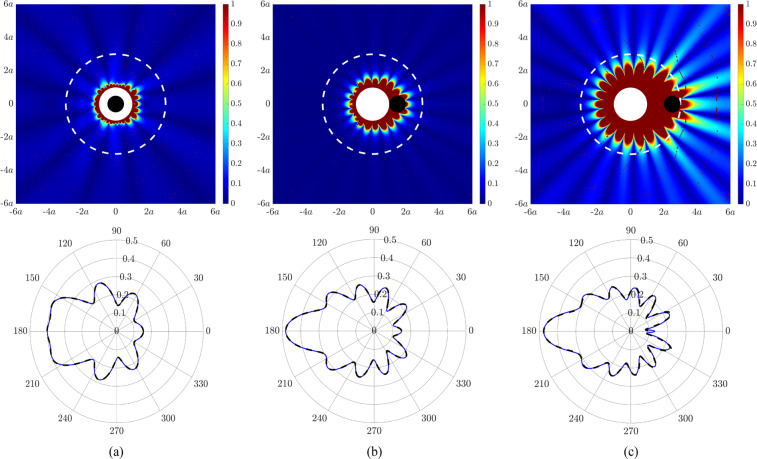
(Top row) Deviation (in dB) of the actively controlled acoustic field with respect to the desired illusional field. The original acoustic field corresponds to an elastic cylindrical shell (solid white circle) of radius *a* under monopole source excitation at 283.4 Hz. The desired illusional fields correspond to the scattered acoustic field due to a rigid cylinder of radius $0.5a$ (**a**) concentrically located with the elastic cylindrical shell, (**b**) at a radial distance of $1.5a$ in the forward-scatter region, and (**c**) at a radial distance of $2.5a$ in the forward-scatter region. The illusion cylinder is represented by the solid black circle whilst the error sensor perimeter is denoted by the dashed white line. (Bottom row) Corresponding acoustic directivity (in Pascal) for the illusion pressure (solid blue line) and controlled pressure (dashed black line) at a radial distance of 3*a*.

The performance of the proposed technique to actively generate an acoustic illusion is examined. Figure 4 presents the deviation (top row) and acoustic directivity (bottom row) of the actively controlled field with respect to the desired illusional field. The deviation corresponds to the absolute difference between the actively controlled acoustic field (in dB) and the desired acoustic illusion (in dB). The directivity corresponds to the absolute acoustic pressure (in Pascal) evaluated for all field points along the circumference at a specific radial distance. The acoustic field of the original system corresponds to that for a cylindrical shell of radius *a* under monopole excitation at 283.4 Hz, as shown in Fig. 2(a). For the deviation in Fig. 4(a), the desired acoustic illusion corresponds to that for a rigid cylinder of radius $0.5a$ concentrically located with the original cylindrical shell. Similarly, for the deviation in Fig. 4(b,c), the desired acoustic illusion corresponds to that for a rigid cylinder of radius $0.5a$ at a radial distance of $1.5a$ and $2.5a$ in the forward-scatter region, respectively. It is important to note that the error sensors must always fully enclose the boundary of the illusion object and are located at a radial distance of 3*a*. In all cases considered, the deviation between the actively controlled field and desired illusional field beyond the perimeter of the error sensors is less than 1 dB. When the illusion cylinder is completely enclosed by the original cylindrical shell (Fig. 4(a)), the illusional field takes effect beyond the surface of the shell. However, as the illusion cylinder is moved away from the original cylindrical shell (Fig. 4(b,c)), the illusional field takes effect beyond the error sensor perimeter. Furthermore, as the distance between the original and illusion objects increases, the performance of the acoustic illusion decreases, with a corresponding increase in control effort. The control configurations to generate the results in Fig. 4(a–c) utilised 11 forces and 18 error sensors, 18 forces and 33 error sensors, 20 forces and 78 error sensors, respectively. The acoustic directivity for each case shown in the bottom row of Fig. 4 highlights that there is no noticeable deviation between the actively controlled pressure and desired illusion pressure.

## Discussion

In summary, we present a method whereby the radial motion of an elastic cylindrical shell is actively modified to generate an acoustic illusion. Illusions were generated to resemble the acoustic field due to scattering by a rigid object of different size and location, as well as an illusion associated with acoustic scattering by multiple bodies. A correlation in superior performance of the active structural acoustic illusion with greater proximity between the original and desired objects was observed. Our approach presents a foundation into practical implementation of acoustic illusions and can be employed in applications where masking of unwanted sound or misrepresentation of targets during stealth operations is required.

## Supplementary information

Supplementary Information.

## Data Availability

The datasets generated during and/or analysed during the current study are available from the corresponding author on reasonable request.
