# Supplementary material for: Active structural acoustic illusions
Source: Sci Rep. 2020 Jun 17;10:9772. doi: 10.1038/s41598-020-66398-8 (PMC7300002; doi:10.1038/s41598-020-66398-8)
Supplement: Supplementary file 1 — Supplementary Information. [file 41598_2020_66398_MOESM1_ESM.pdf]

Active structural acoustic illusions

Daniel Eggler and Nicole Kessissoglou

School of Mechanical and Manufacturing Engineering, The University of New South Wales, Sydney 2052, Australia

1. Vibro-acoustic response of an elastic cylindrical shell

2. Acoustic scattering by two rigid cylinders

1. Vibro-acoustic response of an elastic cylindrical shell

We herein present an analytical formulation to describe the vibro-acoustic response of a thin elastic cylindrical shell excited by an incident plane wave, a monopole source or a structural point force. The shell is of radius a (measured to the mid-plane thickness) and thickness h. The shell model adopted here is based on Donnell-Mushtari theory with a modifying operator by Flügge-Byrne-Lur'ye in which the longitudinal displacement of the cylinder is assumed to be constant [1]. Both the exterior and interior fluid media are assumed to be inviscid with density ρ_f and speed of sound c_f.

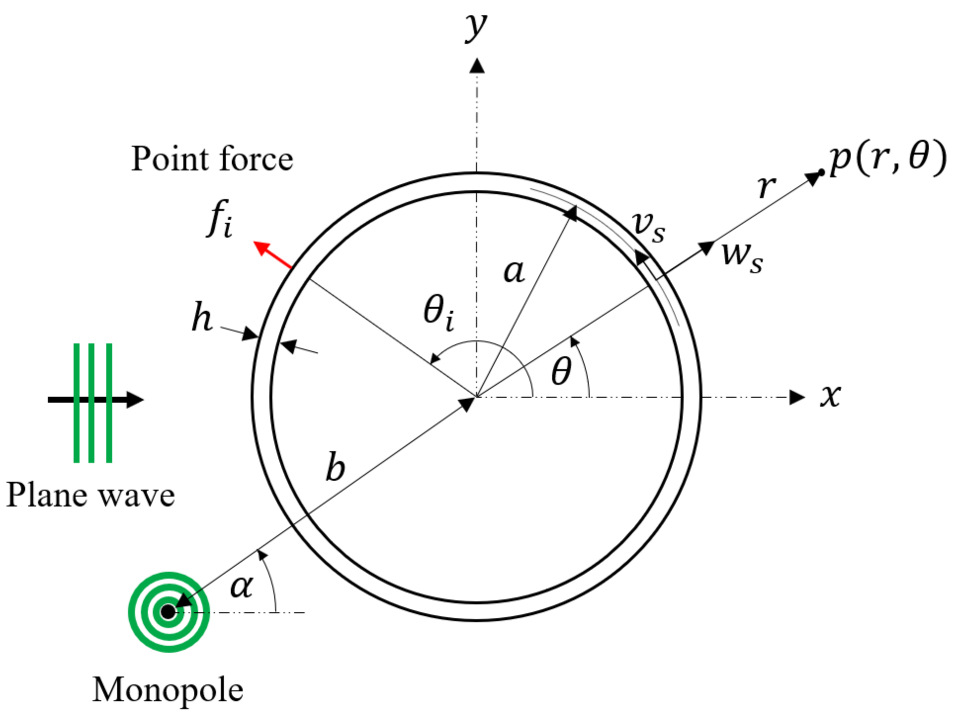

Figure 1. Schematic diagram of the elastic cylindrical shell showing the geometric representation of the system and excitation of the shell by acoustic sources and a localised point force.

Assuming time-harmonic separable solutions for the radial and tangential displacements of the cylindrical shell, denoted by w_s and v_s respectively, the equations of motion are given by [1]

$$\left(D\frac{d^4}{d\theta^4}+D\beta^2-\rho h\omega^2+D\left(1+2\frac{d^2}{d\theta^2}\right)\right)w_s(\theta)+D\beta^2\frac{d}{d\theta}v_s(\theta)=p_{\text{int}}(a,\theta)-p_{\text{ext}}(a,\theta)+f_i(\theta-\theta_i)\,, \quad (1)$$

$$G\frac{d}{d\theta}w_s(\theta)+G\frac{d^2}{d\theta^2}v_s(\theta)+\rho h\omega^2 v_s(\theta)=0\,, \quad (2)$$

where $\beta=\sqrt{12}(a/h)$ is the dimensionless shell thickness parameter, $D=Eh^3/12a^4(1-\nu^2)$ is the flexural stiffness parameter, $G=Eh/a^2(1-\nu^2)$ is the shear stiffness parameter, E is the Young's modulus of elasticity, ρ is the density, ν is Poisson's ratio and ω is the angular frequency. $p_{\text{int}}(r,\theta)$ denotes the acoustic pressure of the interior field, $p_{\text{ext}}(r,\theta)$ is the acoustic pressure of the exterior field, f_i and θ_i respectively denote the amplitude and angular location of the ith localised point force, and $\delta(\theta-\theta_i)$ is the Dirac delta function.

The Helmholtz equation governs the acoustic field. For plane wave excitation, the exterior pressure of the cylindrical shell can be expressed as [2]

$$p_{\text{ext}}(r,\theta)=\sum_{n=-\infty}^{\infty}\left(a_n\frac{J_n(kr)}{J_n'(ka)}+b_n\frac{H_n(kr)}{H_n'(ka)}\right)e^{\text{i}n\theta}\,, \quad (3)$$

where $\text{i}=\sqrt{-1}$, $(\,)'$ is the derivative with respect to the argument, k is the acoustic wavenumber, and J_n, H_n are Bessel and Hankel functions of the first kind of order n, respectively. The first term on the right hand side of Eq. (3) denotes the incident field due to a plane wave, where the coefficient for a plane wave is given by $a_n=\text{i}^n J_n'(ka)$ [2]. The second term on the right hand side of Eq. (3) represents the combined scattered and radiated field, where b_n is an unknown coefficient of order n.

For monopole excitation, the exterior pressure becomes

$$p_{\text{ext}}(r,\theta)=\sum_{n=-\infty}^{\infty}\left(H_0(kR)+b_n\frac{H_n(kr)}{H_n'(ka)}\right)e^{\text{i}n\theta}\,, \quad (4)$$

where R is the distance between the monopole source and the field point (r,θ). Using Graf's addition theorem, the first term on the right hand side of Eq. (4) corresponding to the incident field arising from a monopole source is expressed in terms of the cylinder's local coordinates as follows [2]

$$H_0(kR)=\sum_{n=-\infty}^{\infty}(-1)^n H_n(kb)J_n(kr)e^{-\text{i}n\alpha}\,, \quad (5)$$

where b, α denote the distance and angle between the monopole source and the elastic cylindrical shell, as shown in Fig. 1. The coefficient related to the incident field now becomes

$$a_n = (-1)^n H_n(kb) J_n'(ka) e^{-in\alpha}. \quad (6)$$

For point force excitation, the exterior pressure field corresponds to the structure-borne sound and is given by

$$p_{\text{ext}}(r,\theta) = \sum_{n=-\infty}^{\infty} b_n \frac{H_n(kr)}{H_n'(ka)} e^{in\theta}. \quad (7)$$

For plane wave, monopole or point force excitation of the cylindrical shell, the interior acoustic pressure can be expressed as

$$p_{\text{int}}(r,\theta) = \sum_{n=-\infty}^{\infty} c_n \frac{J_n(kr)}{J_n'(ka)} e^{in\theta}, \quad (8)$$

where c_n is the coefficient for the interior field. The shell radial and tangential displacements are given as follows [1]

$$w_s(\theta) = \sum_{n=-\infty}^{\infty} w_n e^{in\theta}, \quad (9)$$

$$v_s(\theta) = \sum_{n=-\infty}^{\infty} v_n e^{in\theta}, \quad (10)$$

where w_n and v_n are the radial and tangential displacement Fourier coefficients of order n, respectively. The shell radial displacement is coupled to the interior and exterior acoustic pressure fields by the following kinematic conditions

$$\frac{\partial}{\partial r} p_{\text{int}}(a,\theta) = \omega^2 \rho_f w_s(\theta), \quad (11)$$

$$\frac{\partial}{\partial r} p_{\text{ext}}(a,\theta) = \omega^2 \rho_f w_s(\theta). \quad (12)$$

Expressions for the exterior pressure corresponding to Eq. (3) for a plane wave, Eq. (4) for a monopole source or Eq. (7) for point force excitation, the interior pressure given by Eq. (8) and the shell displacements given by Eqs. (9) and (10) are substituted into the equations of motion corresponding to Eqs. (1) and (2) as well as the kinematic conditions given by Eqs. (11) and (12). Orthogonality conditions are then applied to the Fourier series basis $e^{in\theta}$ by multiplying Eqs. (1), (2), (11), (12) by $e^{-im\theta}$ and integrating between 0 and 2π. Finally, the unknown coefficients b_n, c_n, w_n and v_n can be determined, provided the incident field is known or for the case of point force excitation, provided the amplitude and angular location of the point force are known.

2. Acoustic scattering by two rigid cylinders

A brief overview of the analytical framework for acoustic scattering by two rigid cylinders arising from incident plane wave excitation is herein described. Figure 2 shows two rigid cylinders of radii a_1 and a_2, an incident plane wave propagating left to right and the coordinate system used to determine the acoustic pressure at a field point.

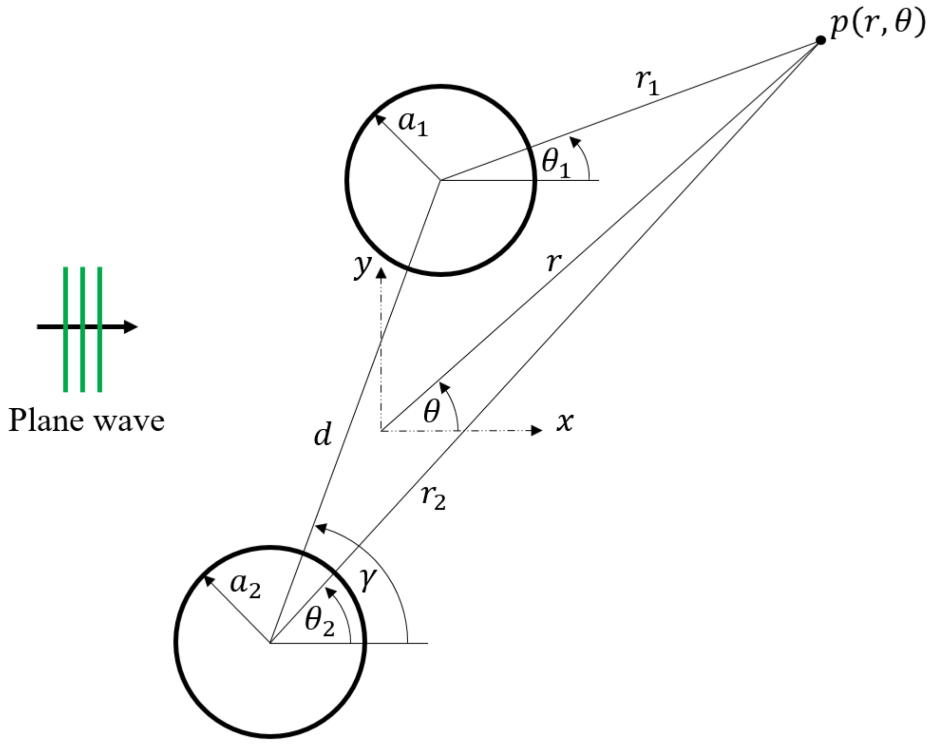

Figure 2. Two arbitrarily located rigid cylinders showing the coordinate system and excitation by an incident plane wave.

In the vicinity of the first cylinder of radius a_1, the total pressure field is given by [2]

$$p(r,\theta) = \sum_{n=-\infty}^{\infty} \left(a_{n,1} J_n(kr_1) e^{in\theta_1} + b_{n,1} H_n(kr_1) e^{in\theta_1} + b_{n,2} H_n(kr_2) e^{in\theta_2} \right), \tag{13}$$

where $p(r,\theta)$ is the acoustic pressure at a point of interest in polar coordinates. The first term on the right hand side of Eq. (13) corresponds to the incident pressure. The second and third terms correspond to the scattered pressure from the first and second cylinders, respectively. $a_{n,1}$ represents the incident field coefficient in the vicinity of the first cylinder, and $b_{n,1}$, $b_{n,2}$ represent the scattered field coefficients associated with the first and second cylinders, respectively. The sound hard boundary condition for the first cylinder is given by

$$\frac{\partial}{\partial r_1} p(a_1, \theta_1) = 0 . \tag{14}$$

Before the boundary condition given by Eq. (14) can be applied, all terms within Eq. (13) must be expressed in terms of the local coordinate system of the first cylinder. Using Graf's addition theorem, the scattered field arising from the second cylinder can be expressed in terms of (r_1, θ_1) and takes the form [2]

$$H_n(kr_2)e^{\mathrm{i}n\theta_2} = \sum_{m=-\infty}^{\infty} S_{nm}(\mathbf{d})J_m(kr_1)e^{\mathrm{i}m\theta_1}\,, \tag{15}$$

where

$$S_{nm}(\mathbf{d}) = H_{n-m}(kd)e^{\mathrm{i}(n-m)\gamma}\,. \tag{16}$$

$\mathbf{d} = (d\cos\gamma, d\sin\gamma)$ is the position vector of the first cylinder of radius a_1 with respect to the second cylinder of radius a_2, and γ is the angle between $\mathbf{d}$ and the x-axis. Substituting Eq. (15) into Eq. (13) yields

$$p(r_1,\theta_1) = \sum_{n=-\infty}^{\infty}\left(a_{n,1}J_n(kr_1) + b_{n,1}H_n(kr_1) + J_n(kr_1)\sum_{m=-\infty}^{\infty} S_{mn}(\mathbf{d})b_{m,2}\right)e^{\mathrm{i}n\theta_1}\,. \tag{17}$$

It is now possible to apply the boundary condition given by Eq. (14). Differentiating Eq. (17) with respect to r_1, evaluating the resulting expression when $r_1 = a_1$ and applying orthogonality conditions for the basis $e^{\mathrm{i}n\theta_1}$ yields

$$b_{n,1}H_n'(ka_1) + J_n'(ka_1)\sum_{m=-\infty}^{\infty} S_{mn}(\mathbf{d})b_{m,2} = -a_{n,1}J_n'(ka_1)\,. \tag{18}$$

The same procedure can be used for the second cylinder of radius a_2, resulting in

$$b_{n,2}H_n'(ka_2) + J_n'(ka_2)\sum_{m=-\infty}^{\infty} S_{mn}(\mathbf{d})b_{m,1} = -a_{n,2}J_n'(ka_2)\,. \tag{19}$$

Equations (18) and (19) form an infinite system of coupled equations from which the solutions for $b_{n,1}$ and $b_{n,2}$ can be obtained provided the incident field is known. For scattering by a single rigid cylinder of radius a, Eq. (19) disappears and Eq. (18) simplifies to the well known expression

$$b_n H_n'(ka) = -a_n J_n'(ka)\,. \tag{20}$$

References

1. Leissa, A. W. *Vibration of shells*. (American Institute of Physics, 1993).
2. Martin, P. A. *Multiple scattering: Interaction of time-harmonic waves with N obstacles*. (Cambridge University Press, 2006).
